# Fungal photoinactivation doses for UV radiation and visible light–a data collection

**DOI:** 10.3934/microbiol.2024032

**Published:** 2024-08-22

**Authors:** Anna-Maria Gierke, Petra Vatter, Martin Hessling

**Affiliations:** Ulm University of Applied Sciences, Department of Medical Engineering and Mechatronics, Albert-Einstein-Allee 55, D-89081 Ulm (Germany)

**Keywords:** *Candida albicans*, *Candida auris*, *Cryptococcus neoformans*, *Aspergillus fumigatus*, *Saccharomyces cerevisiae*, Far-UVC, UVC, UVB, UVA, visible light

## Abstract

Nearly two million people die each year from fungal infections. Additionally, fungal crop infections jeopardize the global food supply. The use of 254 nm UVC radiation from mercury vapor lamps is a disinfection technique known to be effective against all microorganisms, and there are surveys of published UVC sensitivities. However, these mainly focus on bacteria and viruses. Therefore, a corresponding overview for fungi will be provided here, including far-UVC, UVB, UVA, and visible light, in addition to the conventional 254 nm UVC inactivation.

The available literature was searched for photoinactivation data for fungi in the above-mentioned spectral ranges. To standardize the presentation, the mean log-reduction doses were retrieved and sorted by fungal species, spectral range, wavelength, and medium, among others. Additionally, the median log-reduction dose was determined for fungi in transparent liquid media.

Approximately 400 evaluable individual data sets from publications over the last 100 years were compiled. Most studies were performed with 254 nm radiation from mercury vapor lamps on *Aspergillus niger*, *Candida albicans*, and *Saccharomyces cerevisiae*. However, the data found were highly scattered, which could be due to the experimental conditions.

Even though the number of individual data sets seems large, many important fungi have not been extensively studied so far. For example, UV irradiation data does not yet exist for half of the fungal species classified as “high priority” or “medium priority” by the World Health Organization (WHO). In addition, researchers should measure the transmission of their fungal suspensions at the irradiation wavelength to avoid the undesirable effects of either absorption or scattering on irradiation results.

## 1. Introduction

The importance of bacteria and viruses for human health with hundreds of thousands of infections or even fatalities per year worldwide is undisputed [1]–[4]. Besides the treatment of infected patients, the disinfection of air, surfaces, or liquid media by chemical or physical measures plays a major role. Among the physical means is the application of UVC radiation at a wavelength of 254 nm, which acts very efficiently by destroying either the DNA or RNA of various pathogens [5]–[7].

However, the required irradiation doses are not the same for all microorganisms. There can be large differences. For example, vegetative bacterial cells such as *Bacillus subtilis* are much more sensitive than their spores [8]. Tabular overviews exist, which list the common irradiation doses required to reduce known pathogens [8],[9]. However, these tables focus on bacteria and viruses.

The radiation doses necessary for fungi are found only on a much smaller scale, although fungi pose a similar threat to human health as bacteria and viruses. Each year, approximately 150 million fungal infections occur, of which nearly two million are fatal [10]–[13]. Additionally, the World Health Organization (WHO) has recognized the problem of fungal infections, has called for research and action by researchers, and has even published a list of the most significant fungal pathogens [14] similar to the bacterial ESKAPE pathogens [15],[16]. *Cryptococcus neoformans*, *Candida auris*, *Aspergillus fumigatus*, and *Candida albicans* have been identified as particularly important and constitute the “the critical priority group”. Seven additional fungi were named in the next most important “high priority group”, including three more *Candida* species.

In addition to the direct impact of fungi on human health, fungi can also cause other very undesirable effects. It is estimated that fungi are largely responsible for food spoilage [17],[18] and pose a threat to humans in this regard, as well as the annual amount of spoiled food which would have been sufficient to feed 600 million people.

In principle, UVC radiation can be employed as a universal disinfection measure against all fungi via the DNA-destroying mechanism. In the study presented here, the results of already published UVC inactivation studies are compiled and standardized in their presentation. The existing overviews by Kowalski and Malayeri et al. [8],[9] mainly presented data that was obtained with 254 nm UVC radiation from low-pressure mercury vapor lamps. However, other UV spectral ranges also exhibit antimicrobial properties, and the same is true for visible violet or blue light, if the applied dose is high enough [19],[20]. Therefore, in this study, the relevant irradiation range is extended from Far-UVC–starting at 200 nm to visible blue light of wavelengths up to 480 nm.

## 2. Materials and methods

On Pubmed and Google scholar, different combinations of the following terms were searched for: fungi, mold, yeast, inactivation, photoinactivation, reduction, disinfection, antifungal, ultraviolet, UV, UVA, UVB, UVC, Far-UVC, UV-A, UV-B, UV-C, blue light, and violet light. When matching articles were found, the given references were searched for possible further studies. In addition, research was performed to find out which later publications the retrieved paper had cited.

In particular, the mean log-reduction doses were determined for irradiation with either UV or visible light in the spectral range 200–480 nm. If not explicitly stated by the authors themselves, the data were determined from given values or graphs of the respective study as far as possible by determining the mean log-reduction dose from 3 log-reductions. Publications in which either the wavelength or the dose information was either missing or could not be determined were not included. This also applied to experiments within liquid media such as cell culture media or fruit juices, which have very high absorptions, especially in the UV range [21]–[25], and thus prevent the determination of the irradiation dose or only partially irradiate contaminated samples.

Additionally, studies with shorter wavelengths (below 200 nm), longer wavelengths (above 480 nm), very broadband irradiation (>50 nm), or the combination of radiation with other potentially antimicrobial measures including photosensitizers, heat, or extreme pH values were not included. Here, only experiments in the range between 10 and 40 °C and between pH 5 and 8 were included.

When studies investigated different repair mechanisms after irradiation, the data of cultivation in the dark were selected. Studies on particularly radiation-sensitive or -insensitive fungal mutations were not considered.

Then, a categorization was carried out between fungi in liquids, in the air, and on surfaces. Moreover, a distinction was made between vegetative cells, spores, and hyphae. The results were also sorted by spectral range: Far-UVC (200–230 nm), (residual) UVC (230–280 nm), UVB (280–315 nm), UVA (315–400 nm), violet (400–430 nm), and blue (430–480 nm). For each fungus and spectral range, the medians of the log-reduction dose for the liquid samples were determined.

## 3. Results

The literature survey revealed that the study of the disinfecting effect of UV radiation on fungi already started about 100 years ago [26],[27]; for example, it was already recognized at that time that dark/pigmented fungi were relatively resistant to radiation [28] and that experiments that were performed in absorbing cell culture media falsified the measurements [29].

In total, over 100 reports on fungi irradiation were found that met the above criteria. The given or determined individual log-reduction doses can be found in Tables 1 and 2 alongside the obtained medians [for transparent liquids] for different fungi in different spectral ranges. Many investigations were performed on human pathogens, though there were also many plant pathogens and environmental species. The most results were found for *Saccharomyces cerevisiae*, *Candida albicans*, and *Aspergillus niger*.

Over 70% of the individual data sets originated from the UVC spectral range 230–280 nm, which is not surprising since mercury vapor lamps, with their 254 nm emission, are efficient, cheap, easy to use, and have been available for more than 100 years [30].

Table 1 provides the log-reduction doses in the spectral ranges Far-UVC (200–230 nm), (residual) UVC (230–280 nm), and UVB (280–315 nm) in mJ/cm^2^. The antifungal impact of UVA, visible violet, and blue light in Table 2 is several orders of magnitude lower; therefore, the log-reduction doses are given in J/cm^2^.

**Table 1.** Log-reduction doses in mJ/cm^2^ for Far-UVC, UVC and UVB for different fungi and various sample media. Besides the exact wavelength, additional information on strain, medium, temperature, and pH is given, if available.

Fungus	cell type	Far-UVC (200–230 nm) [mJ/cm^2^]	UVC (230–280 nm) [mJ/cm^2^]	UVB (280–315 nm) [mJ/cm^2^]
*Acremonium sp. TC-1-N1-1*	s		***median liquid: 15.4*** 15.4 (254 nm, PBS, [31]);	
*Allescheria boydii*	s		56 (254 nm, agar, [32]);	
	h		28 (254 nm, agar, [32]);	
*Alternaria japonica*	s		5.4 (280, ATCC 44897, air, [33]);	
*Alternaria tenuissima*	s		642 (254 nm, agar, [34]);	
*Aspergillus amstelodami*	s		63.8 (254 nm, air (RH 67%), [35]); 49.8 (254 nm, agar, [35]);	
*Aspergillus awamori*	s		57.6 (254 nm, filter, [36]);	129 (283 nm, filter, [36]);
*Aspergillus brasiliensis*	s		***median liquid: 225*** 225 (254 nm, DSM 1988, water, [37]); 413 (254 nm, DSM 1988, polystyrene, [37]);	
*Aspergillus flavipes*	s		***median liquid: 30.6*** 30.6 (254 nm, liquid, 20 °C, pH 7.9, [38]);	
*Aspergillus flavus*	s		***median liquid: 163.3*** 5.2 (254 nm, JCM 2061, water, [39]); 35.6 (254 nm, liquid, 20 °C, pH 7.9, [38]); 291 (254 nm, KCCM 60330, liquid, [40]); 331 (254 nm, FRR 5660, liquid, [41]); 6.1 (280 nm, ATCC 46110, air, [33]); 35 (254 nm, ATCC 9296, agar, [42]); 85.3 (254 nm, FRR 5660, agar, [41]); 3429 (254 nm, KCCM 60330, round coffee beans, [40]);	
*Aspergillus fumigatus*	s		***median liquid: 16.8*** 3.1 (254 nm, JCM 10253, water, [39]); 30.4 (254 nm, liquid, 20 °C, pH 7.9, [38]); 54 (254 nm, ATCC 14109, agar, [42]); 60.8 (254 nm, agar, [43]); 224 (254 nm, agar, [32]); 2437 (254 nm, ATCC 34506, air filter, [44]);	
*Aspergillus fumigatus*	h + s		***median liquid: 4.6*** 5.8 (255 nm, water, 20 °C, pH 7.3, [45],[46]); 3.3 (265 nm, water, 20 °C, pH 7.3, [46]);	
	h		56 (254 nm, agar, [32]);	
*Aspergillus niger*	s	***median liquid: 72.5*** 25.0 (222 nm, buffered deionized water, [47]); 72.5 (222 nm, IFM 63883, PBS, [48]); 108.3 (222 nm, ATCC 32625, water, [49]);	***median liquid: 107.5*** 4.2 (254 nm, JCM 10254, water, [39]); 26.5 (254 nm, buffered deionized water, [47]); 33.5 (254 nm, liquid, 20 °C, pH 7.9, [38]); 43.1 (254 nm, PBS, [50]); 50.8 (254 nm, IFM 63883, PBS, [48]); 103.8 (254 nm, N402, saline, [51]); 111.1 (254 nm, PBS, [52];) 122.0 (254 nm, ATCC 16404, PBS, [53]); 123.0 (254 nm, ATCC 32625, water, [49]); 241.4 (254 nm, water, [54]); 464.4 (254 nm, liquid, [41]); 1157 (254 nm, CON1 40539, liquid, [55]); 28.5 (265 nm, PBS, [50]); 27.1 (280 nm, PBS, [50]); 359 (254 nm, air [RH 55%], [35]); 12.4 (254 nm, vacuum/filter, [56]); 187 (254 nm, agar, [34]); 189.3 (254 nm, FRR 5664, agar, [41]); 214 (254 nm, cellophane, [57]); 259 (254 nm, agar, [35]); 375 (254 nm, steel, [58]); >448 (254 nm, agar, [32]);	1118 (302 nm, cellophane, [57]); 12000 (313 nm, cellophane, [57]);
*Aspergillus niger*	h + s		***median liquid: 7.3*** 7.3 (265 nm, water, 20 °C, pH 7.3, [45],[46]);	
	h		>448 (254 nm, agar, [32]);	
*Aspergillus parasiticus*	s		***median liquid: 183*** 183 (254 nm, KCCM 60330, liquid, [40]); 6528 (254 nm, KCCM 60330, round coffee beans, [40]);	
*Aspergillus terreus*	s		***median liquid: 13*** 13 (254 nm, liquid, 20 °C, pH 7.9, [38]);	
	h + s		***median liquid: 3.7*** 4.0 (255 nm, water, 20 °C, pH 7.3; [46]); 3.3 (265 nm, water, 20 °C, pH 7.3, [46]);	
*Aspergillus versicolor*	s		***median liquid: 28.2*** 28.2 (254 nm, liquid, 20 °C, pH 7.9, [38]); 16.7 (254, air (RH 85%), [59]); 33.3 (254, air (RH 55%), [59]); 55.2 (254 nm, agar, [43]);	
*Blastocladiella emersonii*	s		4.6 (240 nm, agar, [60]); 3.4 (248 nm, agar, [60]); 2.5 (254 nm, agar, [61]); 2.9 (254 nm, agar, [60]); 2.0 (265 nm, agar, [60]); 2.6 (265 nm, agar, [61]); 1.9 (275 nm, agar, [60]);	2.0 (280 nm, agar, [60]); 5.2 (293 nm, agar, [60]); 12.1 (297 nm, agar, [60]);
*Blastomyces dermatitides*	v		<14 (254 nm, agar, [32]);	
	h		<14 (254 nm, agar, [32]);	
*Botrytis cinerea*	s	3.5 (222 nm, agar, [62]);	***median liquid: 33.1*** 26 (254 nm, MUCL 18864, PBS, pH 7.2, [63]); 40.2 (254 nm, liquid, [64]); 2.1 (254 nm, agar, [62]);	***median liquid: 109*** 109 (302 nm, liquid, [64]);
*Candida albicans*	v	***median liquid: 9.9*** 9.6 (222 nm, NBRC 1385, PBS, [48]); 9.9 (222 nm, DSM 1386, liquid, [65]); 10.4 (222 nm, ATCC MYA-273, PBS, 37 °C, [66]); 4.9 (222 nm, agar, [67]); 8.6 (222 nm, ATCC 10231, glass, [68]); 7.4 (233 nm, agar, [67]);	***median liquid: 9.0*** 6.4 (254 nm, CEC 749, PBS, [69]); 7.6 (254 nm, DSM 1386, liquid, [65]); 8.0 (254 nm, 207 (wt), saline, 25° C, [70]); 8.0 (254 nm, 526 (wt), saline, 25° C, [70]); 8.2 (254 nm, 792 (wt), saline, 25° C, [70]); 8.3 (254 nm, ATCC 18804, water, [71]); 9.7 (254 nm, ATCC 10231, water, [71]); 11.8 (254 nm, NBRC 1385, PBS, [48]); ≤18 (254 nm, PBS, [72]); 21.1 (254 nm, saline, [73]); 30.7 (254 nm, ATCC 10231, water, [54]); 44.7 (254 nm, ATCC 10231; saline, [74]); 283 (272 nm, SC 5314, PBS, [75]); 2.4 (275 nm, ATCC 90028, liquid, [76]); 2.2 (254 nm, ATCC 18804, surface, [77]); 9.3 (254 nm, agar, [67]); 14.0 (254 nm, ATCC 90028, agar, [78]); 21.1 (254 nm, agar, [43]); 28 (254 nm, agar, [32]); 29 (254 nm, ATCC 90028, biofilm on polymethylmethacrylate, [79]); 183 (254 nm, glass, [80]); 217 (254 nm, ATCC 10231, agar, [81]); 3200 (254 nm, CEC 749, wound, [69]); 78.3 (255 nm, ATCC 10231, agar, [82]); <300 (272 nm, liquid, [75]); <500 (272 nm, different surfaces, [75]); 82.3 (275 nm, ATCC 10231, agar, [82]);	862 (“UVB“, H29, agar, [83]);
*Candida auris*	v	4.3 (222 nm, DSM 21092, PBS, [84]);	***median liquid: 14.5*** 14.5 (252 nm, ATCC MYA-5001, PBS, [85]); 6.1 (254 nm, DSM 21092, PBS, [84]); 13.2 (254 nm, ARB 0381, water, [71]); 18.1 (254 nm, ARB 0385, water, [71]); 22.1 (254 nm, ARB 0382, water, [71]); 17.7 (261 nm, ATCC MYA-5001, PBS, [85]); ≤18 (254 nm, PBS, [72]); 7.9 (270 nm, ATCC MYA-5001, PBS, [85]); 11.2 (279.5 nm, ATCC MYA-5001, PBS, [85]);	51.3 (302 nm, DSM 21092, PBS, [84]);
*Candida davisinia*	v		20 (254 nm, agar, [86]);	
*Candida glabrata*	v		***median liquid: 10.4*** 2.8 (275 nm, ATCC MYA-2950, liquid, [76]); ≤18 (254 nm, PBS, [72]);	
*Candida guilliermondii*	v		***median liquid: 35*** 35 (254 nm, liquid, [87]);	***median liquid: 3850*** 3850 (313 nm, liquid, [87]);
*Candida krusei*	v		***median liquid: 2.4*** 2.4 (275 nm, ATCC 6258, liquid, [76]); 26.2 (255 nm, ATCC 6258, agar, [82]); 63.9 (275 nm, ATCC 6258, agar, [82]);	
*Candida parapsilosis*	v	4.7 (222 nm, agar, [67]); 7.8 (233 nm, agar, [67]);	***median liquid: ≤18*** ≤18 (254 nm, PBS, [72]); 5.8 (254 nm, agar, [67]);	
*Candida sp (similar to Candida pomicola)*	v		***median liquid: 11.9*** 11.9 (254 nm, PYCC 5991, water, [88]);	
*Candida tropicalis*	v		***median liquid: ≤18*** ≤18 (254 nm, PBS, [72]);	
*Candida utilis*	v		***median liquid: 40.3*** 36.5 (254 nm, ATCC 9950, water, 25 °C, [89]); 44 (254 nm, liquid, [87]);	***median liquid: 3350*** 3350 (313 nm, liquid, [87]);
*Cephalosporium sp*.	h		28 (254 nm, agar, [32]);	
*Cladosporium cladosporiodes*	s	21.2 (222 nm, DSM 19653, PBS, [84]);	***median liquid: 169.3*** 44.1 (254 nm, DSM 19653, PBS, [84]); 52.6 (254 nm, water, [31]); 286 (254 nm, water, [54]); 368 (254 nm, liquid, [64]); 100 (254 nm, NBRC 30313, agar, [90]); 313 (254 nm, agar, [43]); 750 (254 nm, steel, [58]); 20.9 (275 nm, KTC 26803, agar, 27 °C, [91]);	***median liquid: 493*** 435 (302 nm, DSM 19653, PBS, [84]); 550 (302 nm, liquid, [64]);
*Cladosporium halotolerans*	s		90,0 (252, ATCC 10391, metal, [92]); 88.9 (261, ATCC 10391, metal, [92]); 66.7 (270, ATCC 10391, metal, [92]); 67.2 (280, ATCC 10391, metal, [92]);	
*Cladosporium herbarum*			***median liquid: 288*** 288 (254 nm, liquid, [64]); 35.9 (254 nm, air (RH 53%), [35]); 23.9 (254 nm, agar, [35]);	***median liquid: 307*** 307 (302 nm, liquid, [64]);
*Cladosporium trichoides*	s		112 (254 nm, agar, [32]);	
	h		56 (254 nm, agar, [32]);	
*Cladosporium wernecki*	s		448 (254 nm, agar, [32]);	
	h		56 (254 nm, agar, [32]);	
*Colletotrichum acutatum*	v	2.7 (222 nm, agar, [62]);	1.4 (254 nm, agar, [62]); 2.9 (254 nm, JN 543063, lupin seeds, [93]);	
*Colletotrichum fioriniae*	s	1.5 (222 nm, F44, agar, [62]);	6.6 (254 nm, F44, agar, [62]);	
*Colletotrichum gloeosporioides*	s	3.6 (222 nm, CG 162, agar, [62]); 1.0 (222 nm, GMAL 4049, agar, [62]);	4.1 (254 nm, CG 162, agar, [62]); 5.1 (254 nm, GMAL 4049, agar, [62]);	
*Colletotrichum nymphaeae*	s	< 1.5 (222 nm, SL 566, agar, [62]);	6.2 (254 nm, SL 566, agar, [62]);	
*Colletotrichum sp*.	s	2.5 (222 nm, SK-1, agar, [62]);	3.9 (254 nm, SK-1, agar, [62]);	
*Cryptococcus carnescens*	v		***median liquid: 14.5*** 14.5 (254 nm, PYCC 5988, water, [88]);	
*Cryptococcus neoformans*	v	27 (222 nm, var grubii, glass, [68]);	***median liquid: 45.8*** 45.8 (254 nm, ATCC B3501, liquid, [94]); 2.4 (254 nm, KN99α, surface, [77]); 14.4 (254 nm, ATCC 24067, agar, [95]); 28 (254 nm, agar, [32]);	
*Cryptococcus terricola*	v		17 (254 nm, agar, [86]);	
*Cryptococcus victoriae*	v		12 (254 nm, agar, [86]);	
*Curvularia lunata*	h		56 (254 nm, agar, [32]);	
*Epidermophyton floccosum*	s		***median liquid: 26.3*** 6.3 (254 nm, water, 30 °C, [96]); 46.2 (254 nm, PBS, [97]); <14 (254 nm, agar, [32]);	
	h		<14 (254 nm, agar, [32]);	
*Eurotium rubrum*	s		***median liquid: 125.5*** 125.5 (254 nm, FRR 5666, liquid, [41]); 43.4 (254 nm, FRR 5666, agar, [41]);	
*Exophiala xenobiotica*	v		20 (254 nm, agar, [86]);	
*Fusarium graminearum*	s		90.1 (254 nm, DAOM 178148, agar, [98]); 77.0 (277 nm, DAOM 178148, agar, [98]);	
*Fusarium oxysporum*	s		***median liquid:* 38.4** 38.4 (254 nm, ATCC 36576, liquid, [99]);	
*Fusarium solani*	s		***median liquid: 34.9*** 34.9 (254 nm, Saccardo, liquid, [99]);	
*Fusarium sp*.	s		56 (254 nm, agar, [32]);	
	h		112 (254 nm, agar, [32]);	
*Geotrichum candidum*	v		17.3 (254 nm, agar, [43]);	
*Giberella fujikuroi*	s		56 (254 nm, agar, [32]);	
	h		56 (254 nm, agar, [32]);	
*Glomerella cingulata*	s		***median liquid: 24.8*** 24.8 (254 nm, liquid, [100]);	
*Hormondendrum pedrosoi*	s		56 (254 nm, agar, [32]);	
	h		28 (254 nm, agar, [32]);	
*Histoplasma capsulatum*	v		<14 (254 nm, agar, [32]);	
	h		<14 (254 nm, agar, [32]);	
*Leucosporidiella muscorum*	v		12 (254 nm, agar, [86]);	
*Malassezia furfur (=Pityrosporum orbiculare)*	v			63.1 (UVB, ATCC 44341, agar, [83]); 87.9 (UVB, ATCC 42132, agar, [83]); 348 (300 nm; ATCC 44341, agar, [83]);
*Melampsora lini*	s		170 (254 nm, agar, [101]);	
*Metschnikowia viticola similar to Candida kofuensis*	v		***median liquid: 9.4*** 9.4 (254 nm, PYCC 5993, water, [88]);	
*Microsporum canis*	s		***median liquid: 20.0*** 20.0 (254 nm, PBS, [97]); <14 (254 nm, agar, [32]);	
	h		<14 (254 nm, agar, [32]);	
*Microsporum gypseum*	s		56 (254 nm, agar, [32]);	
	h		<14 (254 nm, agar, [32]);	
*Monilinia fructigena*	s		***median liquid: 16*** 16 (254 nm, CBS 101499, PBS, pH 7.2, [63]);	
*Mucor mucedo*	s		67.8 (254 nm, air [RH 63%], [35]); 39.9 (254 nm, agar, [35]);	
*Mucor sp*.	s		<14 (254 nm, agar, [32]);	
	h		28 (254 nm, agar, [32]);	
*Neurospora crassa*	c		***median liquid: 16.4*** 17.5 (238 nm, saline, [102]); 16.4 (254 nm, saline, [102]); 9.7 (265 nm, saline, [102]); 15.6 (280 nm, saline, [102]);	31.5 (302 nm, saline, [102]);
*Nocardia asteroides*	h		28 (254 nm, agar, [32]);	
*Penicillium chrysogenum*	s		33.9 (254 nm, air [RH 41%], [35]); 23.9 (254 nm, agar, [35]); 82.7 (254 nm, agar, [43]); 16.4 (275 nm, KTC 6933, agar, 27 °C, [91]);	
*Penicillium commune*	s		300 (254 nm, steel, [58]);	
*Penicillium corylophilum*	s		***median liquid: 160*** 160 (254 nm, FRR 5661, liquid, [41]); 38.1 (254 nm, FRR 5661, agar, [41]);	
*Penicillium digitatum*	s		***median liquid: 40.0*** 40.0 (254 nm, ATCC 10030; liquid, [99]); 19.1 (254 nm, agar, [103]); 19.2 (254 nm, NBRC 33116, agar, [90]); 25.3 (254 nm, orange, [104]); 110.5 (254 nm, orange, [103]);	
*Penicillium expansum*	s	***median liquid: 14.0*** 14.0 (222 nm, ATCC 36200, water, [49]); 1.7 (222 nm, agar, [62]);	***median liquid: 16.3*** 16.3 (254 nm, ATCC 36200, water, [49]); 21.3 (254 nm, P99418, saline, 25 °C, [105]); 15.3 (277 nm, P99418, saline, 25 °C, [105]); 1.0 (254 nm, agar, [62]); 55.2 (254 nm, P99418, apple, 25 °C, [105]); 60.7 (254 nm, CLX 1499, pear, [106]); 66.7 (254 nm, CLX 1499, apple, [107]); 84.0 (254 nm, CLX 1499, strawberry, [107]); 87.5 (254 nm, CLX 1499, cherry, [107]); 118 (254 nm, CLX 1499, raspberry, [107]); 33.5 (277 nm, P99418, apple, 25 °C, [105]);	
*Penicillium italicum*	s		***median liquid: 46.8*** 46.8 (254 nm, ATCC 48814; liquid, [99]); 17.1 (254 nm, agar, [103]); 241 (254 nm, orange, [104]); 420 (254 nm, orange, [103]);	
*Penicillium multicolor*	s		***median liquid: 49.2*** 49.2 (254 nm, water, [54]);	
*Penicillium oxalicum*	s		462 (254 nm, steel, [58]);	
*Penicillium pinophilum*	s		***median liquid: 117.7*** 117.7 (254 nm, NBRC 6345, water, pH 6.7, [108]);	
*Penicillium polonicum*	s		***median liquid: 21.5*** 16.1 (254 nm, water, [31]); 27.1 (254 nm, PBS, [50]); 21.3 (265 nm, PBS, [50]); 21.7 (280 nm, PBS, [50]);	
*Penicilium sp*.	s		***median liquid: 142.3*** 60.5 (254 nm, PBS, [52]); 224 (254 nm, agar, [32]);	
	h		28 (254 nm, agar, [32]);	
*Pestalotiopsis clavispora*	s		***median liquid: 122*** 122 (254 nm, liquid, [64]);	***median liquid: 167*** 167 (302 nm, liquid, [64]);
*Pichia membranaefaciens*	v		***median liquid: 0.18*** 0.15 (266 nm, KCCM 12470, peptone water, 22 °C, [109]); 0.2 (279 nm, KCCM 12470, peptone water, 22 °C, [109]);	
*Puccinia coronata*	s		600 (254 nm, agar, [101]);	
*Puccinia graminis*	s		2400 (254 nm, agar, [101]);	
*Rhizopus oryzae*	s		***median liquid: 12.7*** 12.7 (254 nm, ATCC 9363, liquid, [110]); 31.6 (254 nm, agar, [34]); >448 (254 nm, agar, [32]);	
*Rhodosporodium babjevae*	v		***median liquid: 47.6*** 47.6 (254 nm, PYCC5996, water, [88]);	
*Rhodosporidium kratochvilovae*	v		12 (254 nm, agar, [86]);	
*Rhodotorula minuta*	v		***median liquid: 28.6*** 28.6 (254 nm, PYCC5990, water, [88]);	
*Rhodotorula mucilaginosa*	v		***median liquid: 47.1*** 38.5 (254 nm, PYCC5989, water, [88]); 55.6 (254 nm, PYCC5995, water, [88]); 13.3 (254 nm, agar, [43]);	
*Rhodotorula sp*.	v		112 (254 nm, agar, [32]);	
*Saccharomyces cerevisiae*	v	***median liquid: 5.0*** 5.0 (222 nm, DSM 70449, PBS, [84]); 18.7 (200 nm, wt (diploid), vacuum / filter, [111]); 22.1 (210 nm, C420-3B RAD (wt, haploid), filter, [112]); 14.7 (210 nm, C420-3B RAD (wt, haploid, dried), filter, [112]); 21.6 (220 nm, XS1972 RAD/RAD (wt, diploid), vacuum/filter, [113]); 22.5 (222.5 nm, ATCC 2335, agar, [114],[115]); 12.0 (230 nm, C420-3B RAD (wt, haploid), filter, [112]); 10.8 (230 nm, C420-3B RAD (wt, haploid, dried), filter, [112]);	***median liquid: 12.5*** 2.5 (254 nm, diploid, liquid, [116]); 5.2 (254 nm, wt (diploid), liquid, [117]); 5.4 (254 nm, RC43a (haploid), liquid, [117]); 6.3 (254 nm, NBRC 1046, water, pH 6.7, [108]); 7.1 (254 nm, DSM 70449, PBS, [84]); 8.3 (254 nm, RAD+ (wt), water, [118]); 17.4 (254 nm, “RAD-RAD” (wt/diploid); [119]); 21.2 (254 nm, XS800 (wt, diploid), water, 20 °C, [120]); 30.2 (254 nm, T1 (wt, diploid), [121]); 33 (254 nm, XS800 (wt, diploid), liquid, [122]); 72.9 (254 nm, KE 162, liquid, [123]); 16.7 (266 nm pulsed, wt, PBS, [124]); 38.4 (238 nm, ATCC 2335, agar, [114],[115]); 11.3 (240 nm, C420-3B RAD (wt, haploid), filter, [112]); 3.2 (240 nm, C420-3B RAD (wt, haploid, dried), filter, [112]); 23.1 (248 nm, l ATCC 2335, agar, [114],[115]); 3.7 (250 nm, XS1972 RAD/RAD (wt, diploid), vacuum/filter, [113]); 1.8 (254 nm, C420-3B RAD (wt, haploid, dried), filter, [112]); 3.7 (254 nm, C420-3B RAD (wt, haploid), filter, [112]); 16.7 (254 nm, ATCC 2335, agar, [114], [115]); 48.5 (254 nm, 211-1a (wt, haploid), agar, [125]); 51.1 (254 nm, D7 (diploid), agar, [126]); 3.0 (263 nm, C420-3B RAD (wt, haploid, dried), filter, [112]); 5.5 (263 nm, C420-3B RAD (wt, haploid), filter, [112]); 25.2 (263 nm, 211-1a (wt, haploid), agar, [125]); 15.2 (265 nm, ATCC 2335, agar, [114], [115]); 30.3 (265 nm, D7 (diploid), agar, [126]);	***median liquid: 6842*** 47.8 (302 nm, DSM 70449, PBS, [84]); 6842 (308 nm pulsed, wt 211 (diploid), liquid, [127]); 9220 (UVB, D7 (diploid), water, [128]); 18.5 (280,4 nm, ATCC 2335, agar, [114],[115]); 9.0 (282 nm, C420-3B RAD (wt, haploid), filter, [112]); 2.0 (282 nm, C420-3B RAD (wt, haploid, dried), filter, [112]); 42.9 (283 nm, 211-1a (wt, haploid), agar, [125]); 41.5 (285 nm, D7 (diploid), agar, [126]); 122.9 (293 nm, 211-1a (wt, haploid), agar, [125]); 166 (295 nm, D7 (diploid), agar, [126]); 43.2 (297 nm, C420-3B RAD (wt, haploid), filter, [112]); 11.2 (297 nm, C420-3B RAD (wt, haploid, dried), filter, [112]); 781 (302 nm, ATCC 2335, agar, [114], [115]); 884 [303 nm, 211-1a (wt, haploid), agar, [125]); 7726 (305 nm, D7 (diploid), agar, [126]); 25554 (310 nm, D7 (diploid), agar, [126]);
*Saccharomyces cerevisiae*			34.5 (273 nm, 211-1a (wt, haploid), agar, [125]); 24.8 (275 nm, D7 (diploid), agar, [126]);	14285 (313 nm, 211-1a (wt, haploid), agar, [125]); 9200 (313 nm, C420-3B RAD (wt, haploid), filter, [112]); 621 (313 nm, C420-3B RAD (wt, haploid, dried), filter, [112]);
	s		***median liquid: 5.0*** 5.0 (254 nm, diploid, liquid, [117]);	
*Saccharomyces pastorianus*	v		***median liquid: 0.7*** 1.0 (266 nm, KCCM 11523, peptone water, 22 °C, [109]); 0.4 (279 nm, KCCM 11523, peptone water, 22 °C, [109]);	
*Saccharomycopsis lipolytica*	v		***median liquid: 330.5*** 297 (254 nm, H195-5, saline, [129]); 364 (254 nm, H194-15, saline, [129]);	
*Scopulariopsis brevicaulis*	s		53.8 (254 nm, air [RH 79%], [35]); 41.9 (254 nm, agar, [35]);	
*Sporotrichum schenckii*	v		28 (254 nm, agar, [32]);	
*Stachybotrys chartarum*			572 (254 nm, ATCC 208877, agar, [130]);	
*Torula bergeri*	h		448 (254 nm, agar, [32]);	
*Torula sphaerica*	v		1.4 (254 nm, air [RH 65%], [35]); 14 (254 nm, agar, [35]);	
*Trichoderma harzianum*			***median liquid: 25.0*** 14.3 (254 nm, water, [31]); 30.1 (254 nm, PBS, [50]); 25.5 (265 nm, PBS, [50]); 24.5 (280 nm, PBS, [50]);	
*Trichophyton mentagrophytes*	s		***median liquid: 42.9*** 35.7 (254 nm, PBS, [97]); 50 (254 nm, water, 30 °C, [96]);	
*Trichophyton rubrum*	s	***median liquid: 13.6*** 13.6 (222 nm, IFM 64661, PBS, [48]);	***median liquid: 27.6*** 8.6 (254 nm, IFM 64661, PBS, [48]); 27.6 (254 nm, PBS, [97]); 40.1 (254 nm, water, 30 °C, [96]); 56 (254 nm, agar, [32]);	
	h		56 (254 nm, agar, [32]);	
*Trichophyton schoenleinii*	s		***median liquid: 53.3*** 53.3 (254 nm, water, 30 °C, [96]);	
*Trichophyton tonsurans*	s		***median liquid: 58.7*** 58.7 (254 nm, water, 30 °C, [96]);	
*Trichophyton violaceum*	s		***median liquid: 9.3*** 9.3 (254 nm, water, 30 °C, [96]);	
*Ustilago zeae*	s	1000 (230 nm; glass; [131]);	1330 (240 nm; glass; [131]); 741 (248 nm; glass; [131]); 565 (254 nm; glass; [131]); 112 (254 nm, agar, [32]); 432 (265 nm; glass; [131]); 532 (280 nm; glass; [131]);	1163 (290 nm; glass; [131]); 3322 (298 nm; glass; [131]); 13300 [303 nm; glass; [131]);
	v		112 (254 nm, agar, [32]);	

v: vegetative cells; s: spores including conidia; h: hyphae including mycelium; PBS: phosphate buffered saline; wt: wild-type

**Table 2.** Log-reduction doses in J/cm^2^ for UVA and visible violet and blue light for different fungi and various sample media. Besides the exact wavelength, additional information on strain, medium, temperature, and pH is given, if available.

Fungus	cell type	UVA (315–400 nm) [J/cm^2^]	Violet (400–430 nm) [J/cm^2^]	Blue (430–480 nm) [J/cm^2^]
*Aspergillus flavus*	s		***median liquid: 628*** 628 (405 nm, PBS, [132]);	
*Aspergillus fumigatus*	s		***median liquid: 295*** 295 (405 nm, PBS, [132]); 250 (405 nm, wound, [132]);	
*Aspergillus niger*	s		***median liquid: 438.9*** 438.9 (405 nm, MUCL 38993, PBS, 29 °C, [133]);	
*Candida albicans*	v	9.7 (365 nm, ATCC 90028, agar, [78]); 727 (“UVA“, H29, agar, [83]);	***median liquid: 94.3*** 73.5 (405 nm, liquid, [134]); 115 (405 nm, MUCL 29903, PBS, 29 °C, [133]); 232.3 (405 nm, SN152, PBS, 37 °C, [135]); 13.0 (415 nm, CEC 749, PBS, [136]); 33.3 (405 nm, agar, [137]); 63.3 (405 nm, ATCC 18804, biofilm on resin, [138]); 94.8 (405 nm, ATCC 18804, biofilm on resin, [139]);	1.5 (420 nm, ATCC 90028, agar, [78]); 571 (450 nm, ATCC 10231, agar, [140]); 45.2 (455 nm, ATCC 18804, biofilm on bones, [141]); 99 (460 nm, ATCC 10231, agar, [142]);
*Candida albicans*	v		100.0 (405 nm, ATCC 10231, agar, [140]); 26.0 (406 nm, ATCC 90028, agar, [78]); 109.8 (415 nm, ATCC 10231, agar, [140]); 247 (415 nm, CEC 749, wound, [136]);	
*Candida auris*	v	***median liquid: 77.5*** 77.5 (365 nm, DSM 21092, PBS, [143]); 13 (365 nm, ARB 0381, steel, [144]);	***median liquid: 104.2*** 104.2 (400 nm, DSM 21092, PBS, [143]);	***median liquid: 769*** 769 (450 nm, DSM 21092, PBS, [143]);
*Candida glabrata*	v		94.8 (405 nm, ATCC 90030, biofilm on resin, [139]);	
*Cladosporium cladosporiodes*	s	***median liquid:* 92.6** 92.6 (365 nm, DSM 19653, PBS, [143]); 14.4 (370 nm, KTC 26803, agar, 25.7 °C, [91]); 45.1 (385 nm, KTC 26803, agar, 25.7 °C, [91]);	***median liquid:* 1000** 1000 (400 nm, DSM 19653, PBS, [143]); 54.8 (405 nm, KTC 26803, agar, 25.7 °C, [91]);	***median liquid:* 7992** 7992 (450 nm, DSM 19653, PBS, [143]);
*Fusarium oxysporum*	s		***median liquid:* 443.5** 313 (405 nm, IHEM 25499, PBS, 37 °C, [135]); 574 (405 nm, PBS, [132]);	
*Fusarium solani*	s		***median liquid:* 175.6** 175.6 (405 nm, IHEM 6092, PBS, 37 °C, [135]);	
*Malassezia furfur (Pityrosporum orbiculare)*	v	22.7 (“UVA”, ATCC 44341, agar, [83]); 35.7 (“UVA”, ATCC 42132, agar, [83]); 14.2 (330 nm; ATCC 44341, agar, [83]); 235.3 (360 nm; ATCC 44341, agar, [83]);		
*Penicillium chrysogenum*	s	11.8 (370 nm, KTC 6933, agar, 27 °C, [91]); 39.0 (385 nm, KTC 6933, agar, 27 °C, [91]);	41.1 (405 nm, KTC 6933, agar, 27 °C, [91]);	
*Penicillium digitatum*	s	***median liquid: 56.3*** 56.3 (385 nm, liquid, [145]);	***median liquid: 57.6*** 57.6 (405 nm, liquid, [145]);	
*Penicillium expansum*	s	***median liquid: 127*** 127 (385 nm, liquid, [145]);	***median liquid: 168*** 168 (405 nm, liquid, [145]);	
*(Eu-) Penicillium lapidosum*	v	***median liquid: 90.9*** 90.9 (365 nm, NBRC 6100, liquid, [146]);		
*Rhizopus microsporus*	s		***median liquid: 2274*** 2274 (405 nm, “12.6652333”, PBS, 37 °C, [135]);	
*Saccharomyces cerevisiae*	v	***median liquid: 37.0*** 0.5 (365 nm, X174 (haploid), liquid, [147]); ≤12.5 (355 nm pulsed, wt, PBS, [124]); 37.0 (400 nm, DSM 70449, PBS,[143]); 47.6, (365 nm, NBRC 1136, liquid, [146]); 66 (364 nm (laser), water, [148]);	***median liquid: 62.5*** 62.5 (400 nm, DSM 70449, PBS, [143]); 56 (405 nm, MUCL 28749, PBS, 29 °C, [133]); 182 (405 nm, DSM 70449, PBS, 30 °C, pH 7, [149]);	***median liquid: 596.4*** 526 (450 nm, DSM 70449, PBS, 30 °C, pH 7, [149]); 666.7 (450 nm, DSM 70449, PBS, [143]);
*Scedosporium apiospermum*	s		***median liquid: 154.3*** 154.3 (405 nm, IHEM 14462, PBS, 37 °C, [135]);	
*Scedosporium prolificians*	s		***median liquid: 144.0*** 144.0 (405 nm, IHEM 5608, PBS, 37 °C, [135]);	
*Trichophyton rubrum*	s		***median liquid: <157*** <157 (405 nm, MUCL 38993, liquid, [150]);	

v: vegetative cells; s: spores including conidia; h: hyphae including mycelium; PBS: phosphate buffered saline; wt: wild-type

**Figure 1.**
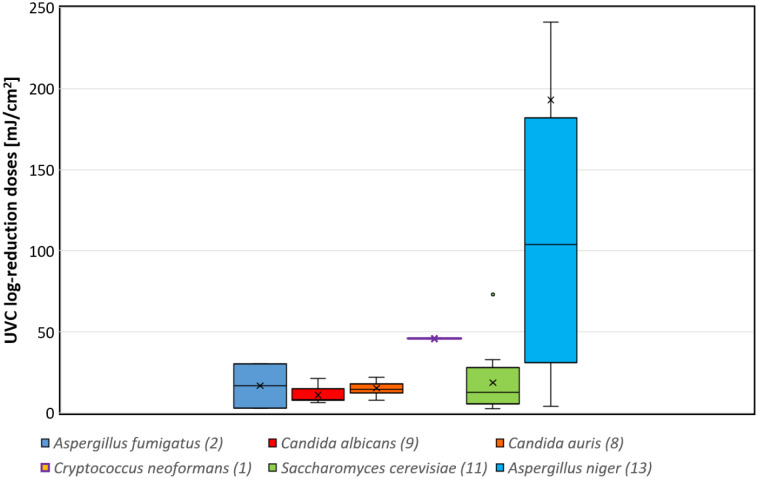
Box-Plots of published fungal UVC log-reduction doses for the WHO “critical priotity group” together with the number of reported single log-reduction doses in brackets. For comparison, the corresponding data for *S. cerevisiae* and *A. niger* (spores) are added. (Two outliers for *A. niger* (spores) are above 250 mJ/cm^2^ and not displayed here.)

The median and average UVC log-reduction doses for fungal suspensions from the WHO “critical priority group”—*A. fumigatus (spores)*, *C. albicans*, *C. auris*, and *C. neoformans*—are also illustrated as boxplots in Figure 1 alongside boxplots for *S. cerevisiae* and *A. niger* (spores) for comparison. Besides *C. neoformans*, the median log-reduction doses in the WHO “critical priority group” are below 20 mJ/cm^2^; the *C. neoformans* value is based on a single investigation. For most members of the “critical priority group”, the median log-reduction doses are in the same order of magnitude as the median log-reduction dose of the non-pathogenic *S. cerevisiae*.

With the help of fungi for which the log-reduction dose medians are available for different spectral ranges, a rough comparison of the antifungal effect of radiation from different spectral ranges can be provided. The determined median far-UVC log-reduction doses are mostly slightly lower than the corresponding log-reduction dose observed with conventional UVC irradiation for the same fungus; however, this statement is based on a rather low number of far-UVC results. No major difference in photosensitivity or log-reduction doses can be observed between both ranges.

In contrast, a comparison between UVC and the visible spectral range displays large differences. The violet log-reduction doses are 3 to 4 orders of magnitude higher than those in the UVC range. On the other hand, the differences between violet and UVA are, in most cases, less than a factor of 2, with *Cladosporium cladosporiodes* (spores) as the only determined exception.

## 4. Discussion

Although Tables 1 and 2 may seem rather lengthy, it can be noted that not much has been studied thus far. For example, UVC data are even missing for half of the fungi named in the WHO “high priority group” and the “medium priority group” [14]–even though inexpensive UVC sources (mercury vapor lamps) have been available for more than one hundred years.

In the other spectral ranges, even less fungal inactivation data have been published, although these ranges are also very interesting and allow for disinfection applications without posing a major hazard to humans. This is true for UVA and visible light [151]; however, the radiation has a strong antimicrobial effect, especially for the far-UVC range, and has been considered to be relatively harmless to humans thus far [152],[153]. Therefore, far-UVC has a great potential to contain the spread of fungi in the future.

The individual values in Tables 1 and 2 displayed a large scatter of the log-reduction doses, even within one species and one wavelength range. For *A. niger*, *C. albicans*, and *S. cerevisiae*, there were 1–2 orders of magnitude between each the smallest and the largest UVC log-reduction dose in the liquid samples.

One reason for this is the biological variations or differences between the individual strains and possibly different physiological states. Another reason is probably the differing experimental set-ups and experimental conditions. One important aspect is the culturing condition after antimicrobial irradiation because illumination can lead to photoreactivation [52],[60],[154]–[156], which results in higher log-reduction doses compared to dark cultivation. As mentioned above, if results of the different illuminations after the antimicrobial irradiation were published, the dark cultivation results were selected. However, in most cases, no statements on the illumination conditions were provided.

Besides this, even for standard irradiation with low-pressure mercury vapor lamps, which all mainly emit at 254 nm, different temperatures, irradiances, and durations have been mentioned. The latter does not lead to major effects due to the Roscoe-Bunsen law; however, there is another very critical point, which, by itself, can lead to variations in the determined log-reduction doses by a factor of 10. As already observed by Coblentz in 1924 [29], and as already mentioned above, absorption [and scattering] in the irradiated medium can lead to lower disinfection success. This would manifest itself, for example, in larger log-reduction doses and a stronger non-mono-exponential behavior. Some authors seem to be aware of the problem [39],[43],[45],[53],[73],[100],[109],[150],[157], though most published studies did not comment on transmission at the irradiation wavelength. This does not only concern the pure medium, but also fungal suspensions. A double-digit number of authors provided cell or spore concentrations of ≥ 10^7^ CFU/mL. In our own (unpublished) measurements on 10^7^ *S. cerevisiae* per mL, we observed an optical density at 600 nm of OD_600_ = 0.3. For 254 nm, the optical density under these conditions was OD_254_ = 1.7. For a path length of 10 mm, this resulted in an irradiance decrease by almost 2 orders of magnitude to about 2% of the initial value. Many authors applied thinner layers of fungal suspensions; however, even behind a 2 mm thin layer, the irradiance would have dropped by about 50%.

## 5. Conclusions

Up to now, the topic of radiation disinfection of fungi did not seem to be of great importance. Even the photoinactivation properties of many health-endangering fungi have been insufficiently studied thus far. Hopefully, this may now somewhat change with the WHO report on the most dangerous fungi [14]. These should be preferentially examined in detail, and for all fungi-or even all pathogens-the far-UVC range seems particularly promising.

Regarding the implementation of the required irradiation experiments, we would recommend always measuring at least the transmission of the fungal suspension to be irradiated at the respective wavelength and, if possible, to achieve a high transmission of more than 50% better 90%.

## Use of AI tools declaration

The authors declare they have not used Artificial Intelligence (AI) tools in the creation of this article.
